# Prevalence of *Helicobacter pylori* in children in eastern Turkey and molecular typing of isolates

**DOI:** 10.1590/S1517-838246220140234

**Published:** 2015-06-01

**Authors:** Gokben Ozbey, Yasar Dogan, Kaan Demiroren, Ibrahim Hanifi Ozercan

**Affiliations:** 1 Firat University, Vocational School of Health Services, Firat University, Elazig, Turkey, Vocational School of Health Services, Firat University, Elazig, Turkey.; 2 Firat University, Department of Pediatric Gastroenterology, Faculty of Medicine, Firat University, Elazig, Turkey, Department of Pediatric Gastroenterology, Faculty of Medicine, Firat University, Elazig, Turkey.; 3 Firat University, Department of Pathology, Faculty of Medicine, Firat University, Elazig, Turkey, Department of Pathology, Faculty of Medicine, Firat University, Elazig, Turkey.

**Keywords:** children, culture, *Helicobacter pylori*, PCR-RFLP

## Abstract

The objectives of the present study were to determine *Helicobacter pylori* via culture, polymerase chain reaction and histopathological diagnosis in 101 children ranging in age from 4 to 18 years, to identify the association among restriction fragment length polymorphism types and clinical disease and to investigate the relationships among different isolates of *H. pylori* in different age groups. We observed a high prevalence of *H. pylori* infections in children between the ages of 13 and 18 (75.8%), while children aged 4 to 6 years had the lowest prevalence of infection (40%). *H. pylori* was detected in 30.7% (31 of 101), 66.3% (67 of 101) and 63.2% (60 of 95) of children as determined by culture methods, PCR and histological examination, respectively. *H. pylori* isolates with RFLP types I and III were the most common among children with antral nodularity, whereas RFLP types II and IV were the least detected types. Interestingly, all isolates from peptic ulcer patients were type III. Although our results show a high prevalence of *H. pylori* infections in the pediatric population in eastern Turkey, no association was identified between *H. pylori* infection with antral nodularity and recurring abdominal pain. In addition, we found low genetic variation among *H. pylori* isolates from children and no association between RFLP types and antral nodularity (p > 0.05). Additionally, we found that *H. pylori* isolates with specific RFLP types were predominant in different age groups.

## Introduction

Most *Helicobacter pylori* infections are thought to be acquired in childhood or adolescence, and infection with this bacterium at a young age increases the risk of associated complications later in life (Vinette *et al.*, 2004).

Culture methods have been the "gold standard" for the detection of bacterial pathogens, yet for bacteria such as *H. pylori*, this technique is often difficult and time-consuming (Singh *et al.*, 2008). Serological tests also have limitations such as low specificity and failure to differentiate between active and past infections (Hestvik *et al.*, 2010). Polymerase chain reaction (PCR)-based techniques have successfully been used to detect pathogens that may be difficult to culture, identify and/or isolate from clinical samples (Singh *et al.*, 2008). PCR-based restriction fragment length polymorphism (RFLP) analysis, used in this study, has sufficient discriminatory power to differentiate among *H. pylori* strains, in addition to being a relatively simple, fast and low cost sub-typing method (Andreson *et al.*, 2007; Li *et al.*, 1997).

Currently, little is reported about the prevalence of active *H. pylori* infections among children in the Elazig Province of eastern Turkey. The aims of this study were to: (i) identify the prevalence of active *H. pylori* infection among children and determine if prevalence differs with age, (ii) evaluate any correlation between *H. pylori* infection and gastroduodenal disease and (iii) determine if *H. pylori* RFLP sub-types are associated with antral nodularity, peptic ulcer, specific age groups and/or clinical outcomes.

## Materials and Methods

### Patients

A total of 101 patients were enrolled in this study, including 53 girls and 48 boys ages 4 to 18 years (Ozbey *et al.*, 2013). Symptoms included recurrent abdominal pain, vomiting with or without blood, bloody stools and growth retardation. The children underwent endoscopy at the clinic of Pediatric Gastroenterology Department at the Firat University Hospital in the period of March 2011 to September 2012 (Ozbey *et al.*, 2013) Ethical clearance for this study was provided by the Medical Ethics Committee of Firat University. Informed consent was obtained from each patient and signed by the children's parents prior to the endoscopy procedure.

### Bacterial culture

Bacterial culturing of the antral biopsy was performed as described elsewhere (Chomvarin *et al.*, 2006). Briefly, the antral biopsies were placed directly into sterile Eppendorf tubes containing 0.5 mL of Brain Heart Infusion broth (Oxoid, Basingstoke, UK) with 15% glycerol and processed for culture within 2 h. Each sample was smeared onto Columbia agar base (Oxoid, UK) added with 7% laked horse blood (SR0048C, Oxoid, UK) and *H. pylori* Dent's supplement (Oxoid, UK). Plates were incubated at 37 °C in a microaerobic atmosphere using the Campygen gas generating kit (Oxoid, UK) for up to 10 days (Frenck *et al.*, 2006). Typical small, round colonies that were gram negative and urease, catalase and oxidase positive were presumed to be *H. pylori* (Goodwin and Wesley, 1993). All isolates were stored at −80 °C in Brain Heart Infusion broth added with 15% glycerol until further analysis. Reference *H. pylori* strains, including some clinical strains, were provided by the Department of Medical Biology, Faculty of Medicine, Pamukkale University, Denizli, Turkey. The histological evaluation of each antral biopsy sample was conducted by a pathologist according to the Sydney classification system (Dixon *et al.*, 1996).

### Primers and PCR conditions

DNA from antral biopsy samples and suspensions of *H. pylori* colonies were purified using the QIAamp DNA mini kit (Qiagen, Germany). The forward [*glm*M-F (5′-AAGCTTTTAGGGGTGTTAGGGGTTT-3′)] and reverse [*glm*M-R (5′-AAGCTTACTTTCTAACACTAAC GC-3′)] primers (Lu *et al.*, 1999) amplify a region of the *glm*M gene (formerly *ure*C) of *H. pylori* to yield a 294 bp PCR product. The thermal cycling was as follows: 35 cycles of denaturation at 93 °C for 1 min, 1 min at an annealing temperature of 55 °C, and a 1 min extension step at 72 °C (Lu *et al.*, 1999).

### PCR-based amplification and RFLP analysis of PCR amplicons

For PCR-RFLP analysis, the *ureC* gene was amplified using the forward *ure*C-U (5′- AAG AAG TCA AAA ACG CCC CAA AAC -3′) and reverse *ure*C-L (5′- CTT ATC CCC ATG CAC GAT ATT CCC -3′) primers to yield a PCR product size of 1169 bp (Li *et al.*, 1997). The PCR cycling consisted of the following steps: a denaturation step at 94 °C for 5 min, 45 cycles at 94 °C for 45 s, 59 °C for 30 s, 72 °C for 1 min 30 s, and a final extension step at 72 °C for 10 min (Andreson *et al.*, 2007). Ten microliters of PCR product was restricted with the restriction enzyme *Hha*I (Fermentas, Lithuania) according to the manufacturer's instructions.

### Statistical analysis

Statistical analysis was performed using the statistical software program SPSS 12.00 (SPSS, Chicago, IL, USA). Relationships among the prevalence of *H. pylori* in children and the RFLP types with clinical outcomes were analyzed using Fisher's exact and Pearson's χ ^2^ tests. A p value < 0.05 is statistically significant.

## Results

Of the 101 patients analyzed, 58 (57.4%) were experiencing abdominal pain, 22 (21.8%) had growth retardation, 12 (11.9%) had bloody vomit and/or blood in stools, 8 (7.9%) were vomiting, and 1 (1%) was diagnosed with anemia. Endoscopic findings revealed antral nodularity, antral hyperemia, hyperemia in duodenal mucosa, duodenal ulcers and gastric ulcers in 54.5%, 12.9%, 23.8%, 3% and 5.9% of cases, respectively.

### Culture and PCR results

**Figure 1:**
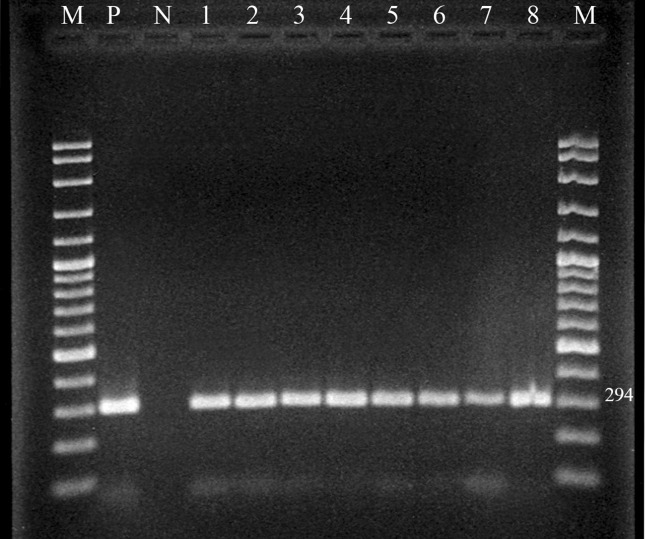
An agarose gel of PCR products of *H. pylori* isolates from antral biopsy specimens (M: DNA Ladder (100 bp), P: *H. pylori* positive control, N: negative control, 1–8: *H. pylori* positive samples).

*Helicobacter pylori* was detected in antral gastric biopsies by culture, PCR and histology in 30.7%, 66.3% and 63.2% of patients, respectively (Table 1). All *H. pylori* isolates examined generated the expected 294 bp fragment of the *glm*M gene (Figure 1). Patients 13 to 18 years of age showed the highest prevalence of infection (75.8%), while children 4 to 6 years of age had the lowest prevalence (40%) (Table 2). There was no statistically significant difference in the prevalence of *H. pylori* between males (66.7%, 32/48) and females (67.9%, 36/53). *H. pylori* was detected by PCR in 76.4% (42/55) of cases presenting with antral nodularity, 46.2% (6/13) presenting with antral hyperemia, 50% (12/24) presenting with hyperemia in duodenal mucosa, 66.7% (2/3) presenting with duodenal ulcers and 83.3% (5/6) presenting with gastric ulcers (Table 3). Statistical analysis was not performed because the number of cases for a particular symptom or diagnosis was relatively small. However, the number of cases of recurring abdominal pain and antral nodularity were relatively high. *H. pylori* was detected in 68.9% (40/58) of cases reporting recurring abdominal pain, but no correlation was found between the prevalence of *H. pylori* infection with recurring abdominal pain and that with antral nodularity.

**Table 1:** Prevalence of *H. pylori* as determined by culture, PCR and histopathological findings*.*

Tests	*H. pylori* (+) n (%)	*H. pylori* (−) n (%)	Total (n)
culture	31 (30.7)	70 (69.3)	101
PCR	67 (66.3)	34 (33.7)	101
Histopathology	60 (63.2)	35 (36.8)	95

**Table 2:** *H. pylori* prevalence according to different age groups.

Years	*H. pylori* (+) n (%)	*H. pylori* (−) n (%)	Total (n)
4–6	2 (40)	3 (60)	5
7–12	40 (63.5)	23 (36.5)	63
13–18	25 (75.8)	8 (24.2)	33

**Table 3:** Prevalence of *H. pylori* isolates in children according to endoscopic findings as detected by PCR.

Endoscopic findings	*H. pylori* (+)n (%)	*H. pylori* (−)n (%)
Antral nodularity (n = 55)	42 (76.4)	13 (23.6)
Antral hyperemia (n = 13)	6 (46.2)	7 (53.8)
Hyperemia in duodenal mucosa (n = 24)	12 (50)	12 (50)
Duodenal ulcer (n*=*3)	2 (66.7)	1 (33.3)
Gastric ulcer (n*=*6)	5 (83.3)	1 (16.7)
Total (101)	67 (66.3)	34 (33.7)

n: number of *H. pylori* isolates.

### Histopathology results

Six cases were excluded because of insufficient tissue for histopathological examination. Of the remaining 95 antrum biopsy samples, histological examination showed that 60 (63.2%) were positive for *H. pylori.* Pathological analysis of the biopsy material showed that intestinal metaplasia was present in only a single child (a 13-year old male) who also had chronic gastritis and was *H. pylori* positive*.* None of the cases presented with gastric atrophy.

### RFLP analysis results

**Figure 2:**
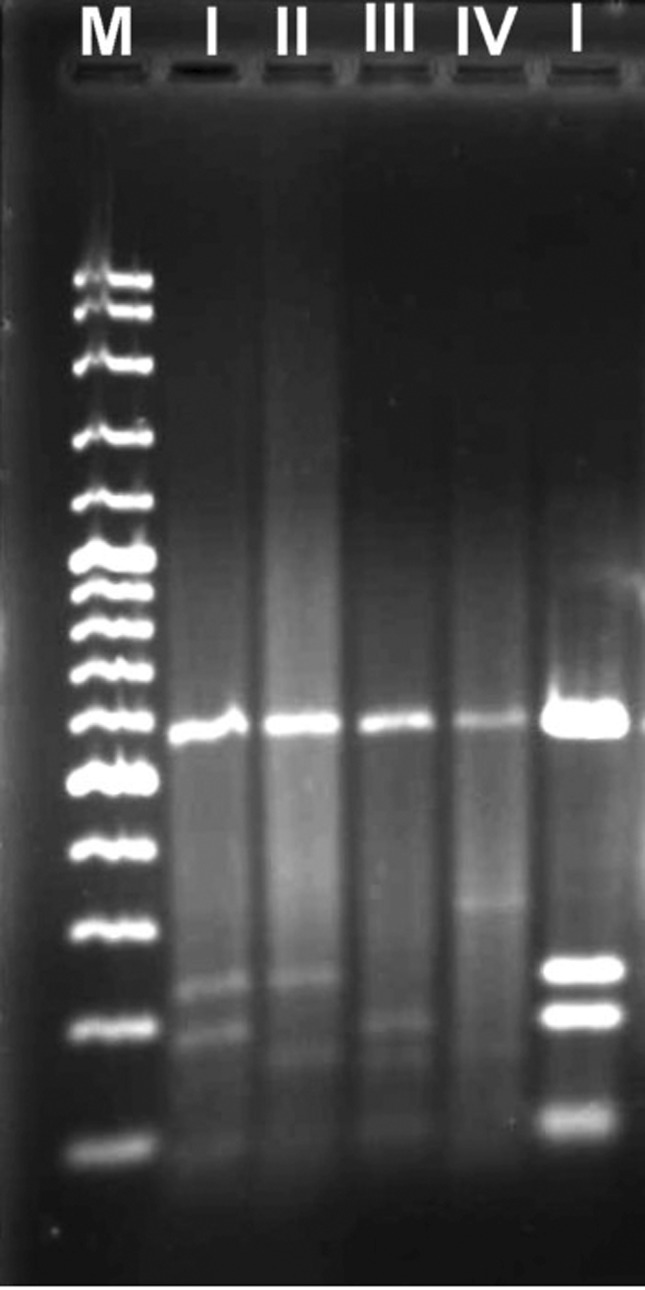
PCR-RFLP analysis of *H. pylori* isolates using restriction enzymes in agarose gel (2.5% w/v) (M: 100 bp DNA ladder, lanes I, II, III and IV: RFLP band profiles).

PCR amplification of *H. pylori* DNA using the *ureC* primer set produced an amplicon of the expected size (1169 bp) for all *H. pylori* isolates examined. The RFLP analysis of our isolates revealed a low heterogeneity among isolates. Table 4 shows the distribution of *H. pylori* RFLP types. Four profiles (types I, II, III and IV) were identified (Figure 2). From the cases with antral nodularity, types I (44.8%) and III (27.6%) were the predominant strains while types II and IV were detected at in lower numbers (10.4% and 17.2%, respectively). From cases presenting with peptic ulcers, only type III *H. pylori* was detected.

**Table 4:** RFLP types of 31 clinical *H. pylori* isolates obtained after digestion with *HhaI* enzyme.

RFLP types	Antral nodularity n (%)	Peptic ulcer n (%)	Total n (%)
I	13 (44.8)	-	13 (41.9)
II	3 (10.4)	-	3 (9.7)
III	8 (27.6)	2 (100)	10 (32.3)
IV	5 (17.2)	-	5 (16.1)
Total	29	2	31

n: number of *H. pylori* isolates.

Only two isolates from culture were obtained from the 4 to 6 year old age group; one was RFLP type I and the other type II. In the 7 to 12 year old age group (17 isolates positive by culture), 47% of isolates were type I (8/17), 5.9% type II (1/17), 35.3% type III (6/17) and 11.8% type IV (2/17). In addition, both types I and III were detected in 33.3% (4/12) of 13 to 18 year olds. Likewise, types II and IV were detected in the same percentage of children (16.7%, 2/12) within this age group (Table 5). However, due to the relatively small number of children with peptic ulcers, the statistical analysis required to associate RFLP types with this medical condition could not be performed. No relationship was identified between RFLP type and antral nodularity in the present work (p > 0.05).

**Table 5:** RFLP types of 31 *H. pylori* isolates determined after culturing and grouped according to patient age.

RFLP types	4–6 n (%)	7–12 n (%)	13–18 n (%)
I	1 (50)	8 (47)	4 (33.3)
II	-	1 (5.9)	2 (16.7)
III	-	6 (35.3)	4 (33.3)
IV	1 (50)	2 (11.8)	2 (16.7)
Total	2	17	12

n: number of *H. pylori* isolates.

## Discussion

Acquiring knowledge on *H. pylori* infections is important for human pathogen studies because the lack of current knowledge is hindering actions to protect human health. Although RFLP analysis of *H. pylori* isolates in adults has been performed in eastern Turkey (Ozbey *et al.*, 2012), there are currently no studies that report the isolation and genotyping of *H. pylori* in children residing in this same region. The current study was undertaken to examine the prevalence and genetic diversity of *H. pylori* isolates from children in the Elazig Province of Turkey. These results suggest that appropriate and effective treatment strategies should be implemented against *H. pylori* infections in the future.

The majority of studies investigating the prevalence of *H. pylori* among children in Turkey have employed serological methods (Selimoglu *et al.*, 2002; Yucel *et al.*, 2009). Other studies show varied prevalence of *H. pylori* in asymptomatic children including 7.1% in the Czech Republic (0–15 year olds), 24.7% in Israel (0–5 year olds), 30.9% in Turkey (2–12 year olds), 31.6% in Portugal (0–15 year olds) and 82% in Iran (0–15 year olds) (Alborzi *et al.*, 2006; Kori *et al.*, 2009; Oleastro *et al.*, 2011; Sykora *et al.*, 2009; Yucel *et al.*, 2009). We found that the highest prevalence of infection was found in children from 13 to 18 years of age (75.8%). Our study also showed that a total of 66.3% of symptomatic children were infected with *H. pylori* as detected by PCR. A lower prevalence of *H. pylori* in children with dyspepsia (38%) was reported in the United States (Sood *et al.*, 2005). However, our findings match previous data reported by Selimoglu *et al.* (2002) in randomly selected healthy children between the ages of 6 and 17 in Turkey (64.4% infected). This higher *H. pylori* prevalence may be due to sociodemographic factors such as low socioeconomic status, poor sanitary conditions, higher percentage of low-income families, higher percentage of parents with a low educational background, poor living conditions, high density living quarters and higher rates of immigrant children from the surrounding cities (Azevedo *et al.*, 2009).

Culture-positive biopsies were relatively low at 30.7% (31/101) as compared to histological and PCR results but were similar to the number of positive biopsies detected in Turkey's neighbor, Iran, which reported a *H. pylori* prevalence of 39.8% using the culture method (Iranikhah *et al.*, 2013). Negative culture results may be due to low colonization because of patchy localization of bacteria in the stomach, decreased bacterial viability, overgrowth of potential contaminants, technical problems during tissue transport, increased transportation times and varied culturing methods (Ozcay *et al.*, 2004; Torres *et al.*, 2001).

In this study, most of the children reported recurrent abdominal pain, but the role of *H. pylori* in causing chronic abdominal pain is still disputable (Zeyrek *et al.*, 2008; Hestvik *et al.*, 2010). Our data showed no significant correlation between abdominal symptoms and *H. pylori* infection.

Bahu *et al.* (2003) noted a significant relationship between *H. pylori* and endoscopic nodular gastritis. Here, antral nodularity was observed in 55 children (54.5% of those tested) of which 76.4% were positive for *H. pylori*. This observation is similar to previous data (73.8%) reported by Dogan *et al.* (2007) but higher than that reported in Turkey (64.7%) by Ozcay *et al.* (2004) and lower than reported in Japan where 98.5% of *H. pylori* cases were associated with antral nodularity (Kato *et al.*, 2004).

Reports show that the peptic ulcer prevalence in children in different countries varies between 1.8% to 19.5% (Elitsur and Lawrence, 2001; Kato *et al.*, 2004). In the present study, 8.9% of symptomatic children had peptic ulcers, similar to results reported by Megraud (2005) in European children (8.6%) but lower than those reported by Ugras and Pehlivanoglu (2011) in Turkish children (13.2%). However, in the current study, the prevalence of *H. pylori* in patients also presenting with ulcers was 66.7% for those with duodenal ulcers and 83.3% for those with gastric ulcers. The percentage for those with duodenal ulcers is lower than the earlier reports from Japan and Turkey where 83% and 76.9%, respectively, of patients with duodenal ulcers were positive for *H. pylori* (Kato *et al.*, 2004; Ugras and Pehlivanoglu, 2011). In this study, a similar number of children with gastric ulcers tested positive for *H. pylori* (83.3%), as was reported (85.2%) previously in Turkey (Ugras and Pehlivanoglu, 2011).

In areas with high incidence rates of gastric cancer, gastric atrophy is common among *H. pylori*-infected children (Ricuarte *et al.*, 2005). The prevalence of gastric atrophy varies between countries, and it has been shown that intestinal metaplasia alters with respect to geographic/genetic origins and environmental factors (Kato *et al.*, 2006: Pacifico *et al.*, 2010; Ricuarte *et al.*, 2005; Tutar *et al.*, 2009; Usta *et al.*, 2004). Only one of the 95 symptomatic children (1.1%) had intestinal metaplasia and was positive for *H. pylori*. These data are in concordance with a previous report(Usta *et al.*, 2004) that found intestinal metaplasia in only one of 175 Turkish children infected with *H. pylori*. However, only 4.6% of children in Japan with intestinal metaplasia were positive for *H. pylori* (Kato *et al.* 2006). In contrast, the prevalence of gastric atrophy is high in Columbian children (16%) and much higher in Japanese children (51.9%) (Kato *et al.*, 2006; Ricuarte *et al.*, 2005).

PCR-RFLP has been frequently used for genotyping and discrimination of *H. pylori* strains because it is low cost, rapid, easy to perform and can be used to analyze *H. pylori* genotype diversity (Röesler *et al.*, 2009). The two restriction endonucleases *Hha*I and *Mbo*I were chosen for RFLP analysis of the *ure*C gene (Li *et al.*, 1997). RFLP analysis of the PCR products of our *H. pylori* isolates yielded four different RFLP types (I, II, III and IV). RFLP types I and III were the most frequently detected among children with antral nodularity whereas RFLP types II and IV were detected less frequently. Furthermore, type III was the most predominant type in isolates from patients with peptic ulcers. Our findings confirmed the results of Mishra *et al.* (2002), Saribasak *et al.* (2004) and Kulsantiwong *et al.* (2012) who reported no correlation between the RFLP patterns of all strains and the patients' clinical outcome. In addition, certain RFLP profiles predominated according to different age groups; types I and IV (50%) in 4–6 year olds, type I (47%) in 7–12 year olds and types I and III (33.3%) in 13–18 year olds. These percentages show that children within different age groups were infected with various *H. pylori* strains, which might be related to differences in immune responses towards *H. pylori* during childhood development. Further study is needed to better establish this relationship.

High genetic variation in *H. pylori* strains isolated from various patients has been demonstrated worldwide (Raymond *et al.*, 2005; Kulsantiwong *et al.*, 2012; Menoni *et al.*, 2013). This result is in contrast to the current study where *H. pylori* isolates from children showed a considerably low genetic diversity. However, limited PCR-RFLP profiles might be due to the small numbers of *H. pylori* isolates in the present study (31 total isolates).

In conclusion, the results of the current study show a high prevalence of *H. pylori* infection in children in eastern Turkey. Although the number of *H. pylori* isolates is too small to perform any epidemiological analysis, the bacterial isolates from children in the present study yielded a relatively small number of genetically distinct RFLP types. A different distribution of RFLP types was evident among children of different age groups. Therefore, it is necessary to conduct future work on larger populations of children in order to fully confirm the results of this study and to identify the genetic heterogeneity of *H. pylori* isolates in children from the Elazig Province.
